# Risk Factors for Falls in Community-Dwelling Older Adults During the Novel Coronavirus Pandemic in Japan: A Prospective Cohort Study

**DOI:** 10.3390/ijerph21121603

**Published:** 2024-11-30

**Authors:** Akihiko Murayama, Daisuke Higuchi, Kosuke Saida, Shigeya Tanaka, Tomoyuki Shinohara

**Affiliations:** 1 Department of Physical Therapy, Faculty of Rehabilitation, Gunma University of Health and Welfare, Maebashi Plaza Genki 21 6-7F, 2-12-1 Hon-machi, Maebashi-shi 371-0023, Gunma, Japan; 2 Department of Physical Therapy, Faculty of Health Care, Takasaki University of Health and Welfare, 27 Naka Orui-machi, Takasaki-shi 370-0033, Gunma, Japan; higuchi-d@takasaki-u.ac.jp (D.H.); saida@takasaki-u.ac.jp (K.S.); tanaka-s@takasaki-u.ac.jp (S.T.); shinohara-t@takasaki-u.ac.jp (T.S.)

**Keywords:** social distancing, community-dwelling older adults, falls, predictors of falls, remote assessment

## Abstract

This study aimed to test the hypothesis that knowledge derived from indirect assessments can be used to identify fall risk factors during a period of social distancing. A baseline survey of 1953 community-dwelling older adults was conducted in May 2020, with a follow-up survey performed in May 2023 to assess the situation 3 years later. In total, 339 individuals were followed from baseline to follow-up. Baseline age, sex, Questionnaire for Change of Life, Frailty Screening Index, and Questionnaire for Medical Checkup of Old-Old (QMCOO) scores and subscales were used to determine fall predictors. In addition, history of falls in the past year was assessed at follow-up (outcome). The participants were categorized into fall (n = 78) and non-fall (n = 261) groups. Using binary logistic regression analysis, items that showed significant differences in a between-group comparison were analyzed, and age and history of falls, which were sub-items of the QMCOO, were identified as predictors of falls. Although special assessments may be required during periods of social distancing, we believe that it is important for these assessments to continue being performed as they are performed during normal times.

## 1. Introduction

Falls are a prevalent phenomenon among older adults, with high prevalence rates of fragility fractures and impaired functional capacity [1,2,3]. It is estimated that one to three older adults experience falls at least once annually. Falls account for 5–10% of all fractures, with 1–2% of proximal femoral fractures [4,5]. Consequently, with the increasing life expectancy and proportion of individuals aged ≥65 years, falls have become a prominent global health concern [6].

The social and economic consequences of falls are significant, with fall-related expenditures accounting for approximately 1% of all healthcare expenditures in high-income countries [7]. Accordingly, falls and their associated injuries are becoming progressively prevalent, highlighting the urgent need to develop effective global prevention and management strategies [8,9]. Therefore, identifying at-risk individuals is essential for early intervention to minimize healthcare costs and optimize the quality of life [10]. The Global Burden of Disease Study revealed that falls increased the global burden of disease by 3% over 30 years between 1990 and 2019 [11]. Furthermore, the most recent Global Burden of Disease Study conducted between 2010 and 2021 revealed that falls ranked among the top 25 of the 371 diseases included. Thus, falls are a significant factor influencing the number of years spent below the ideal life expectancy [12].

In Japan, the term “older adults” is used to describe individuals aged ≥65 years. As of 17 September 2024, the older adult population in Japan reached a record high of 36.25 million, accounting for 29.3% of the total population. Furthermore, based on data from 200 countries and regions, Japan has the highest proportion of older adults in the world [13]. A 2022 survey conducted by the Ministry of Health, Labor, and Welfare revealed that with an aging population, falls and fractures have become the third most common cause of older adults requiring nursing care [14]. This represents a notable increase in rank from the fourth position, as reported in a large-scale survey conducted in 2019 [15]. Furthermore, the aforementioned Ministry of Health, Labor, and Welfare survey period, spanning 2019–2022, coincided with the coronavirus disease 2019 (COVID-19) pandemic. However, during these 4 years, limited knowledge regarding fall risk assessments and interventions among community-dwelling older adults was available.

In Japan, voluntary restrictions on activities, such as not going out to prevent the spread of COVID-19, lasted approximately 3 years, from April 2020 to May 2023. During this period, people were compelled to cancel social activities that brought them together. These changes in participation in social activities may have resulted in the emergence or exacerbation of frailty in community-dwelling older adults. Furthermore, there is concern that an increase in the prevalence of coronavirus frailty [16], which could be conceptualized as a secondary consequence of the ongoing pandemic, may occur.

Shinohara [17] conducted a health survey during the COVID-19 pandemic in some areas of Takasaki City, Gunma Prefecture, from 2020 to 2021. This cohort study (hereafter, the Takasaki Survey) was important because it was conducted at the beginning of the spread of the virus in Japan. The objective of the Takasaki Survey was to identify effective strategies to prevent frailty by screening community-dwelling older adults who are believed to require significant support and implementing measures that address specific aspects of their lives. A sub-analysis of the data from the Takasaki Survey was conducted with a focus on the risk factors associated with falls in community-dwelling older adults [18]. In the Takasaki survey, a follow-up survey was conducted in May 2023 to ascertain the actual situation 3 years post-COVID-19. A systematic review indicated that frail older adults are more likely to fall than robust older adults [19]. Therefore, we expect that the assessment of the fall risk using a frailty assessment scale will reduce the burden on both patients and assessors. We also suggest that remote assessment without physical measurements is a potential alternative when in-person assessment is impossible [20,21]. The Takasaki survey used the Questionnaire for Change of Life (QCL), the Frailty Screening Index (FSI), and the Questionnaire for Medical Checkup of Old-Old (QMCOO) as assessment scales that do not require actual measurements to predict frailty in community-dwelling older adults [17]. Therefore, this study aimed to examine whether the scales used in the Takasaki Survey to predict frailty also contributed to the prediction of fall risk factors.

In Japan, there is an urgent need to elucidate, treat, and implement measures against COVID-19. However, we also feel that we should not forget to assess the risk of falls among older adults living in unique circumstances. Therefore, we compared two groups of community-dwelling older adults (fallers and non-fallers) in terms of baseline characteristics and using an assessment scale to predict frailty during the pandemic. Two hypotheses were developed and tested. Hypothesis 1: new risk factors for falls emerged during the COVID-19 pandemic compared with the pre-pandemic period. Hypothesis 2: risk factors for falls identified pre-pandemically should be considered during the COVID-19 pandemic. This study aimed to substantiate these hypotheses and gain new insights into fall prevention among community-dwelling older adults.

## 2. Materials and Methods

### 2.1. Study Design and Participants

Takasaki City has a population of 368,869, with 29.2% of the population aged ≥65 years. Takasaki is the most populous of the 35 municipalities in Gunma Prefecture, and its aging rate is approximately equivalent to the national average. However, this survey is not exhaustive in scope. This prospective cohort study participants were community-dwelling older adults aged ≥65 years who lived in Takasaki City, Gunma Prefecture, and accepted regular home visits from civil welfare commissioners. Civil welfare commissioners, who regularly visited the homes of the study participants, distributed a questionnaire, a document explaining the study, and a questionnaire to potential participants at 6-month intervals.

**Figure 1:**
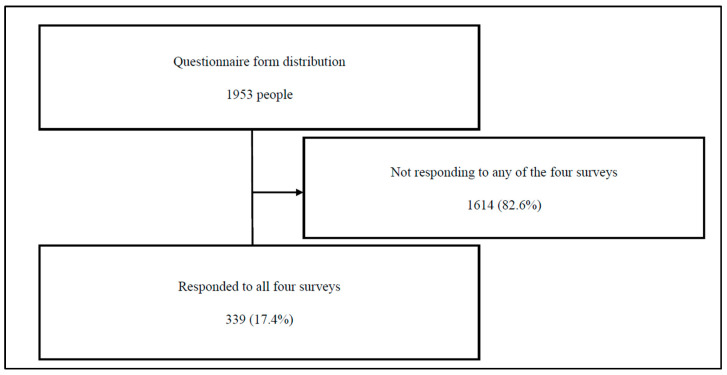
Flowchart of participant selection.

The initial phase of the study involved the administration of a baseline survey to 1953 community-dwelling older adults. The study population comprised 339 individuals, identified by tracing the initial baseline survey to the subsequent follow-up survey (Figure 1). The baseline survey was conducted between 11 May 2020 and 10 July 2020. The secondary survey was conducted between 11 November 2020 and 10 January 2021. The tertiary survey was conducted between 11 May 2021 and 10 July 2021. Furthermore, a follow-up survey was conducted between 10 May and 10 July 2023 to determine the actual situation 3 years after the baseline survey.

The civil welfare commissioners, who conducted regular home visits to the study participants, distributed the questionnaires and research instructions to prospective participants. Individuals who expressed an interest in participating in the study were requested to return the questionnaires and consent forms for research participation via mail. The survey materials were distributed via mail, and the participants were contacted by telephone to provide explanations and confirm the safety of the study. By requiring the participants to return the questionnaires via mail, the research team members ascertained which individuals had completed and returned the questionnaires and, then, aggregated the data.

This study followed the Declaration of Helsinki and was designed in accordance with the ethical guidelines outlined in the “Ethical Guidelines for Medical Science Research Involving Human Subjects”. This study was approved by the Research Ethics Committee of Takasaki University of Health and Welfare (approval numbers 2009 and 2259) and registered with the University Hospital Medical Information Network (UMIN000040335). The research participants were provided with a questionnaire that outlined the purpose and content of the study as well as contact information for any inquiries. To confirm their consent to participate, the participants provided their names in the questionnaire.

### 2.2. Measurements

The survey items included baseline demographic data (age and sex) along with the FSI [22], QMCOO [23], and QCL scores. The questionnaires were designed to be completed in approximately 10 min. At the time of the baseline survey, Japan was in a state of emergency because of the COVID-19 pandemic. This entailed a series of directives, including instructions for citizens to remain at their places of residence, requests for workers to work from home, and the closure of educational institutions, public spaces, and commercial facilities. Accordingly, the survey items were refined with a focus on frailty, which was the primary objective of the Takasaki Survey. The predictive values of baseline age, sex, QCL items, FSI score, QMCO score, and fall sub-items were investigated. In the follow-up survey, a history of falls in the past year was examined as an outcome.

The FSI was assessed using a self-administered questionnaire comprising yes/no answers to five questions. Scores ≥ 3 points indicated the presence of frailty, whereas scores of 1–2 indicated pre-frailty. FSI studies have tracked changes in frailty in older adults, and the FSI was used to accurately identify frailty in older Japanese individuals [24]. The Japanese version of the Cardiovascular Health Study criteria [25] is used to evaluate frailty in Japan, including walking speed and grip strength measurements. This study used the FSI, which is a questionnaire-based alternative that does not require actual measurements.

The QMCOO comprises 15 questions that were described in detail [23]. Previous research has proposed a 0 or 1 scale for the 15 items, defining frailty by a QMCOO score of ≥4 [26]. In Japan, the Kihon Checklist is a valuable tool for identifying individuals with signs of frailty [27]. Nevertheless, because of the considerable time required to answer its 25 questions, we opted to use the QMCOO, which poses a more manageable number of queries.

The QCL comprises five items, to facilitate independent responses by older adults. The items were set based on the amount of activity related to physical frailty and lower limb strength [28], food intake [29], amount of activity related to mental and psychological frailty and anxiety [30], and opportunities for interaction related to social frailty [31], and five answer options were provided. All items required responses regarding subjective changes during the period in which society changed due to COVID-19 and in the past month. Subjective changes over the past month were assessed to evaluate the impact of infection spread prevention measures on lifestyle and physical and mental status. Each item was scored on a scale as 1 (increased or stronger), 2 (slightly increased or stronger), 3 (no change), 4 (slightly decreased or weaker), and 5 (decreased or weaker), with the exception of worry and anxiety. The items pertaining to worry and anxiety were scored on a scale as 1 (decreased), 2 (slightly decreased), 3 (no change), 4 (slightly increased), and 5 (increased). Previous studies have demonstrated the validity of most frailty-related QCL items [32,33].

The fall group included those who reported falling during the year prior to the follow-up survey. Baseline demographics, QCL items, baseline FSI and sub-items, and QMCOO scores and sub-items were compared between the fall and the non-fall groups (those who reported not having fallen in the past year in the follow-up survey). Baseline demographics, QCL items, baseline FSI and sub-items, and QMCOO scores, which are fall predictors, were obtained from the baseline survey conducted. Data for classifying the fall and non-fall groups were obtained from the follow-up survey.

### 2.3. Statistical Analyses

Relevant statistical principles were considered and applied in accordance with an established methodology to determine the required sample size for interval estimation of population proportions. A 5% margin of error was established, and a confidence level of 90% was set. Considering the inherent difficulty in predicting population proportions, a value of 50% was proposed for the predicted population proportion. The required sample size was found to be 385. Missing-value information was initially examined using the R package naniar [34]. In instances in which the exclusion of missing values introduced bias into the dataset, we supplemented the missing values using the R package mice. This multiple-assignment method primarily focuses on the completion of missing values in multivariate datasets. The R package naniar [34] was used to determine whether the exclusion of missing values resulted in bias inclusion within the dataset. Accordingly, missing values were subsequently supplemented using multiple-assignment methods (R package mice [35,36]), which require two conditions: the specification of m, which represents pseudo-complete datasets, and a variable substitution method. Herein, the value of m was set to 10, and the method employed was predictive mean matching, which randomly selects and substitutes observations that are in close proximity to the regression values. The results were subsequently summarized in accordance with Rubin’s rule [37]. Subsequently, the Shapiro–Wilk test was used to assess data normality and found that the data from both groups were not normally distributed. Accordingly, the Mann–Whitney U test was used to ascertain whether any discrepancies existed in age, FSI score, and QMCOO score. The chi-square test was used to determine whether any differences existed regarding sex and QCL, FSI, or QMCOO sub-items. The Fisher exact test was employed in instances where the number of anticipated scores was less than five. Forced-entry binomial logistic regression analysis was used to determine the odds ratios (OR) and 95% confidence intervals (CI). We used the presence or absence of falls as the dependent variable, with significant items serving as the independent variables. For forced entry, we evaluated the variance inflation factor and selected non-multicollinearity items. Multivariate analysis was used to identify the independent predictors. Previous studies underscored the “1 in 10” rule for each predictor [38]. In this case, a minimum of 10 participants per predictor variable was included. A domain was automatically classified as having a high risk of bias if the rule of thumb was unsatisfied. Statistical analyses were performed using EZR [39] (EZR on R commander ver. 1.68), with a significance level of 5%.

## 3. Results

Data from 339 participants were analyzed, with 78 (23.0%) participants in the fall group, and 261 (77.0%) in the non-fall group. A between-group comparison showed significant differences in median age (fall/non-fall: 81/79 years, *p* < 0.01), FSI score (fall/non-fall: 1/1, *p* < 0.05), and QMCOO score (fall/non-fall: 4/3, *p* < 0.001; Table 1). In addition, as shown in Table 1, the median and interquartile ranges of the FSI scores for the fall and non-fall groups were identical; however, the range was 0–4 for the fall group and 0–3 for the non-fall group. These results prompted a comparison between the FSI and QMCOO sub-items. Significant differences were observed in the FSI sub-item “Do you think you are walking more slowly than before?” (“score 1” fall/non-fall: 55.1/40.6%, *p* < 0.05; Table 2) and QMCOO items “Do you think you are walking more slowly than before?” (“score 1” fall/non-fall: 55.1/40.6%, *p* < 0.05), “Have you experienced a fall in the past year?” (“score 1” fall/non-fall: 38.5/12.3%, *p* < 0.001), and “Do you go out at least once a week?” (“score 1” fall/non-fall: 11.5/4.2%, *p* < 0.05; Table 3). No significant differences were observed among the five QCL items (Table 4).

Binomial logistic regression analysis was then conducted with the outcome as the objective variable. There were three items whereby significant differences were observed in the QMCOO (one item overlapped with an item in the FSI) and age as a forced entry explanatory variable (adjusted for sex). Considering the number of participants, it was determined that Type I errors could be avoided by conducting a single binomial logistic regression analysis per the FSI and QMCOO sub-item rather than independently. Thus, age (OR 1.06, 95% CI: 1.01–1.12, *p* < 0.05) and the QMCOO subitem “Have you experienced a fall in the past year (score 1)” (OR 4.18, 95% CI: 2.27–7.71, *p* < 0.001) were identified. The model χ^2^ test result showed *p* < 0.001, and the Hosmer–Lemeshow test result showed *p* = 0.396, indicating a good fit (Table 5).

## 4. Discussion

This study compared the risk of falls among community-dwelling older adults during the COVID-19 pandemic with that during a prolonged period of social isolation and normal periods. It analyzed various measurement factors and indicators related to fall risk and prevention strategies. This study highlights the importance of preventive measures and the need to consider potential contributing factors in the prevention of falls. Furthermore, conducting a survey at two different points over a long period provides valuable insights by comparing the usual conditions with those experienced during social isolation. This approach provides recommendations for improving, modifying, and preventing factors that increase the risk of falls in community-dwelling older adults.

In epidemiological studies on falls among community-dwelling older adults in Japan before the COVID-19 pandemic, the incidence of falls ranged from <10% to >20% [40,41]. The fall rate in our study was 23%, which was similar to the pre-COVID-19-pandemic rate. The novelty of this study is that it examined predictors of falls before and after the COVID-19 outbreak. The objective was to formulate and test two hypotheses. The initial hypothesis proposed that novel risk factors for falls emerged during the pandemic compared with the pre-pandemic period. The second hypothesis proposed that, even during the COVID-19 pandemic, the risk factors for falls identified pre-pandemically should be considered. We constructed a logistic regression model to test our hypotheses, and its results support the second hypothesis, indicating that the factors previously identified as predictors of falls among community-dwelling older adults remained relevant during the COVID-19 outbreak.

Fall risks are associated with aging [42]. Therefore, age-specific fall risk assessments and fall prevention interventions are required, and this was relevant even during the COVID-19 pandemic [43]. In the constructed logistic model, we focused on the history of falls in the past year, as it has been determined to be an important fall risk predictor [43,44,45,46,47]. The American Geriatrics Society and the British Geriatrics Society also recommend that all adults aged ≥65 years undergo an annual screening for history of falls or balance problems [48,49]. Additionally, based on our results, a pre-COVID-19-pandemic history of falls could help to predict falls during the COVID-19 outbreak. This may lead to the prevention of falls in community-dwelling older adults, irrespective of changes in social conditions, and help them to avoid fall-induced trauma.

The COVID-19 pandemic restricted physical and social activities for 3 years. Thus, these findings are important for the management of frailty and disability in older adults and for predicting the likelihood of future adverse health events caused by such restrictions [50]. On 8 May 2023, COVID-19 was classified as a Category 5 infectious disease in Japan. The system of various requests and government involvement based on the law was changed to a response based on voluntary efforts while respecting individual choices [51]. However, even after this categorization, there were more than a few cases where healthcare professionals were concerned about patients returning to their previous lifestyle. This is consistent with recent guidelines stating that the goals, values, and preferences of older adults should be considered when developing plans to prevent falls and related injuries [52]. Therefore, we believe that the results of this study will be useful for future studies. In addition, the World Health Organization [53] has mentioned the necessity of accumulating knowledge, including that about the anticipation of pathogens (disease X) that may cause future epidemics or pandemics.

Finally, considering the current situation where the Japanese government is calling for strengthening preventive measures against the next infectious disease crisis [54], we can provide recommendations based on our findings. In the case of falls, which are influenced by various factors, only the factors observed pre-pandemically were extracted rather than new pandemic-specific factors. It is easy to believe that a unique evaluation is required in an emergency; however, we believe that continuing to conduct evaluations similar to those conducted pre-pandemically is important for a better comprehension of fall prevention and its risks.

Notably, this study has certain limitations. First, the survey response rate was only 17.4%, which precluded any investigation or analysis of the reasons for nonresponses. Herein, only a limited number of participants were followed. Therefore, it is unclear whether the examined cohort with a median age of 80 years and a low male ratio is representative of Takasaki City, Gunma Prefecture, which hinders the generalizability of our findings. If follow-up surveys are continued, it will be necessary to devise measures, such as re-contacting subjects, to encourage the participants to respond. Second, because the survey was not comprehensive, concerns regarding sampling bias cannot be overlooked. Finally, ensuring an adequate sample size is essential for quantitative research. Considering the findings of this study, conducting a similar investigation using a larger sample size to ensure more precise results is advisable. Thus, considering these factors is essential.

We believe that there is a need to identify the risk factors for falls in community-dwelling older adults and establish methods for assessment and intervention [55,56], including new remote exercise intervention approaches that would contribute to fall prevention. Additionally, the results of this study may be relevant for future infectious disease crises.

Despite these research limitations, evidence from prospective cohort studies conducted since the beginning of the COVID-19 outbreak to examine the predictors of falls in community-dwelling older adults is limited. The fact that even during a 3-year emergency with limited survey items we could suggest the utility of assessments similar to those used in the pre-pandemic period in predicting falls demonstrates their significance.

## 5. Conclusions

The novelty of this study is that, by surveying two different time points over a long period, valuable insights were gained by comparing the usual state and that experienced during social isolation. This study identified factors that should be considered in future social policy interventions for fall prevention in community-dwelling older adults to aid in determining whether community-dwelling older adults affected by different lifestyles exhibit similar fall risk factors and health characteristics. Although special assessments may be required during periods of social distancing, we believe that it is important for these assessments to continue being performed as they are performed during normal times.

## Figures and Tables

**Table 1:** Group comparisons of age and sex, along with total FSI and QMCOO scores.

	Overall	Fall Group	Non-Fall Group	*p* Value
	(n = 339)	(n = 78)	(n = 261)	
Age median (interquartile range)	80	81	79	0.007
	(75–83)	(76–85)	(75–82)	
Sex, (male/female), n (%)	73(21.5)/266 (78.5)	15 (19.2)/63 (80.8)	58 (22.2)/203 (77.8)	0.684
FSI (score) median (interquartile range)	1	1	1	0.024
	(0–2)	(0–2)	(0–2)	
QMCOO (score) median (interquartile range)	3	4	3	<0.0001
	(2–4)	(2–4)	(1–4)	

Age, FSI score, and QMCOO score: Mann–Whitney U test; Sex: chi-square test.

**Table 2:** Group comparisons of FSI sub-items.

No.	Questions	Overall (n = 339)	Fall Group (n = 78)	Non-Fall Group (n = 261)	*p* Value
Q1	Have you lost 2–3 kg or more over the past 6 months? (score 0/1), n (%)	309 (91.2)/30 (8.8)	70 (89.7)/8 (10.3)	239 (91.6)/22 (8.4)	0.786
Q2	Do you think you walk more slowly than before? (score 0/1), n (%)	190 (56.1)/149 (43.9)	35 (44.9)/43 (55.1)	155 (59.4)/106 (40.6)	0.032
Q3	Do you do perform physical exercise like walking at least once a week? (score 0/1), n (%)	270 (79.7)/69 (20.3)	62 (79.5)/16 (20.5)	208 (79.7)/53 (20.3)	1.000
Q4	Can you recall what happened 5 min ago? (score 0/1), n (%)	320 (94.4)/19 (5.6)	70 (89.7)/8 (10.3)	250(95.8)/11 (4.2)	0.051
Q5	Have you felt tired for no reason (in the past 2 weeks)? (score 0/1), n (%)	286 (84.4)/53 (15.6)	61 (78.2)/17 (21.8)	225 (86.2)/36 (13.8)	0.126

Sub-items of the frailty screening index: Q1, 2, 3, and 5: chi-square test; Q4: Fisher exact test.

**Table 3:** Group comparisons of QMCOO sub-items.

No.	Questions	Overall (n = 339)	Fall Group (n = 78)	Non-Fall Group (n = 261)	*p* Value
Q1	How is your health condition? (score 0/1), n (%)	309 (91.2)/30 (8.8)	71 (91.0)/7 (9.0)	238 (91.2)/23 (8.8)	1.000
Q2	Are you satisfied with your daily life? (score 0/1), n (%)	307 (90.6)/32 (9.4)	69 (88.5)/9 (11.5)	238 (91.2)/23 (8.8)	0.615
Q3	Do you eat three times a day? (score 0/1), n (%)	322 (95.0)/17 (5.0)	73 (93.6)/5 (6.4)	249 (95.4)/12 (4.6)	0.555
Q4	Do you have any difficulties eating tough foods compared to 6 months ago? (score 0/1), n (%)	244 (72.0)/95(28.0)	54 (69.2)/24 (30.8)	190 (72.8)/71 (27.2)	0.637
Q5	Have you choked on your tea or soup recently? (score 0/1), n (%)	261 (77.0)/78(23.0)	54 (69.2)/24(30.8)	207 (79.3)/54(20.7)	0.088
Q6	Have you lost 2 kg or more in the past 6 months? (score 0/1), n (%)	309 (91.2)/30 (8.8)	70 (89.7)/8 (10.3)	239 (91.6)/22 (8.4)	0.786
Q7	Do you think you walk more slowly than before? (score 0/1), n (%)	190 (56.1)/149 (43.9)	35 (44.9)/43 (55.1)	155 (59.4)/106 (40.6)	0.032
Q8	Have you experienced a fall in the past year? (score 0/1), n (%)	277 (81.7)/62 (18.3)	48 (61.5)/30(38.5)	229 (87.7)/32 (12.3)	<0.0001
Q9	Do you go for a walk for your health at least once a week? (score 0/1), n (%)	270 (79.7)/69 (20.3)	62 (79.5)/16 (20.5)	208 (79.7)/53 (20.3)	1.000
Q10	Do your family or your friends point out your memory loss? For example, you ask the same question over and over again (score 0/1), n (%)	321 (94.7)/18(5.3)	72 (92.3)/6 (7.7)	249(95.4)/12 (4.6)	0.265
Q11	Do you find yourself not knowing today’s date? (score 0/1), n (%)	268 (79.1)/71 (20.9)	61(78.2)/17 (21.8)	207 (79.3)/54 (20.7)	0.958
Q12	Do you smoke? (score 0/1), n (%)	322 (95.0)/17(5.0)	73 (93.6)/5 (6.4)	249 (95.4)/12 (4.6)	0.555
Q13	Do you go out at least once a week? (score 0/1), n (%)	319 (94.1)/20(5.9)	69 (88.5)/9 (11.5)	250 (95.8)/11(4.2)	0.025
Q14	Do you keep regular communication with your family and friends? (score 0/1), n (%)	321 (94.7)/18(5.3)	74 (94.9)/4(5.1)	247 (94.6)/14(5.4)	1.000
Q15	When you are not feeling well, do you have anyone you can talk with? (score 0/1), n (%)	320 (94.4)/19(5.6)	73(93.6)/5(6.4)	247 (94.6)/14(5.4)	0.779

Sub-items of the QMCOO: Q1, 2, 4, 5, 6, 7, 8, 9, 11: chi-square test; Q3, 10, 12, 13, 14, 15: Fisher exact test.

**Table 4:** Group comparisons for QCL sub-items.

No.	Questions	Overall	Fall Group	Non-Fall Group	*p* Value
		(n = 339)	(n = 78)	(n = 261)	
Q1	Amount of daily movement, median (interquartile range)	2	2	2	0.293
		(1–3)	(1–3)	(1–3)	
Q2	Leg muscle strength, median (interquartile range)	3	3	3	0.754
		(2–3)	(2–3)	(2–3)	
Q3	Meal size, median (interquartile range)	3	3	3	0.820
		(3–4)	(3–4)	(3–4)	
Q4	Worry or anxiety, median (interquartile range)	3	3	2.5	0.097
		(2–3)	(2–3)	(2–3)	
Q5	Opportunities to talk to people, median (interquartile range)	3	3	3	0.779
		(3–3)	(3–3)	(3–3)	

Q1–5: Mann–Whitney U test.

**Table 5:** Results of binominal logistic regression analysis of FSI and QMCOO sub-items.

Independent Variable	Odds Ratio	95% Confidence Interval	*p* Value
Age	1.06	1.01–1.12	0.002
Do you think you walk more slowly than before? (score 1)	1.24	0.71–2.17	0.446
Have you experienced a fall in the past year? (score 1)	4.18	2.27–7.71	<0.0001
Do you go out at least once a week? (score 1)	2.17	0.80–5.87	0.128

Binomial logistic regression analysis was performed after the four items with significant differences were forcibly input and adjusted for sex. Hosmer–Lemeshow goodness of fit test. χ^2^ = 8.387, df = 8, *p* value = 0.396.

## Data Availability

The data supporting the findings of this study are available from the corresponding author [A.M.] upon reasonable request.
